# High Leach-Resistant Fire-Retardant Modified Pine Wood (*Pinus sylvestris L.*) by In Situ Phosphorylation and Carbamylation

**DOI:** 10.1021/acsomega.3c00146

**Published:** 2023-03-14

**Authors:** Chia-feng Lin, Olov Karlsson, Oisik Das, Rhoda Afriyie Mensah, George I. Mantanis, Dennis Jones, Oleg N. Antzutkin, Michael Försth, Dick Sandberg

**Affiliations:** † Wood Science and Engineering, Department of Engineering Sciences and Mathematics, Luleå University of Technology, Forskargatan 1, SE-931 77 Skellefteå, Sweden; ‡ Structural and Fire Engineering, Department of Civil, Environmental and Natural Resources Engineering, Luleå University of Technology, SE-971 87 Luleå, Sweden; § Laboratory of Wood Science and Technology, Department of Forestry, Wood Sciences and Design, University of Thessaly, GR-431 00 Karditsa, Greece; ∥ Department of Wood Processing and Biomaterials, Faculty of Forestry and Wood Sciences, Czech University of Life Sciences Prague, Praha 6-Suchdol, CZ-16521 Prague, Czech Republic; ⊥ Chemistry of Interfaces, Department of Civil, Environmental and Natural Resources Engineering, Luleå University of Technology, SE-971 87 Luleå, Sweden

## Abstract

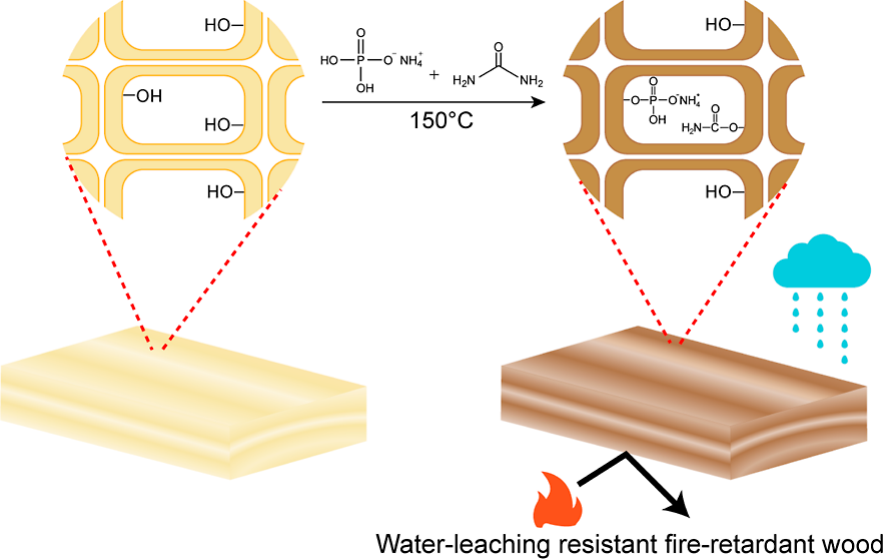

The exterior application of fire-retardant (FR) timber necessitates it to have high durability because of the possibility to be exposed to rainfall. In this study, water-leaching resistance of FR wood has been imparted by grafting phosphate and carbamate groups of the water-soluble FR additives ammonium dihydrogen phosphate (ADP)/urea onto the hydroxyl groups of wood polymers via vacuum-pressure impregnation, followed by drying/heating in hot air. A darker and more reddish wood surface was observed after the modification. Fourier transform infrared spectroscopy, X-ray photoelectron spectroscopy, solid-state ^13^C cross-polarization magic-angle-spinning nuclear magnetic resonance (^13^C CP-MAS NMR), and direct-excitation ^31^P MAS NMR suggested the formation of C–O–P covalent bonds and urethane chemical bridges. Scanning electron microscopy/energy-dispersive X-ray spectrometry suggested the diffusion of ADP/urea into the cell wall. The gas evolution analyzed by thermogravimetric analysis coupled with quadrupole mass spectrometry revealed a potential grafting reaction mechanism starting with the thermal decomposition of urea. Thermal behavior showed that the FR-modified wood lowered the main decomposition temperature and promoted the formation of char residues at elevated temperatures. The FR activity was preserved even after an extensive water-leaching test, confirmed by the limiting oxygen index (LOI) and cone calorimetry. The reduction of fire hazards was achieved through the increase of the LOI to above 80%, reduction of 30% of the peak heat release rate (pHRR_2_), reduction of smoke production, and a longer ignition time. The modulus of elasticity of FR-modified wood increased by 40% without significantly decreasing the modulus of rupture.

## Introduction

Wood is a natural and renewable resource, which has long been considered an important material construction for a sustainable society. Nevertheless, the inherently low biological resistance and durability of species typically used in construction and their relatively low resistance to fire should be addressed in order to comply with modern regulatory requirements for intended applications. Several chemicals, such as acetic anhydride, furfuryl alcohol, dimethylol dihydroxyethylene urea (DMDHEU), phenol-formaldehyde (PF) resin, melamine-formaldehyde (MF) resin, and silicate-/silane-based compounds, have been utilized as wood modification agents for reducing the moisture uptake of treated wood and thereby enhancing its durability.^1^ In general, the chemical modification of wood involves active and/or passive modification. Active modification changes the properties chemically, mainly due to the reaction of hydroxyl groups with the wood polymers, namely, cellulose, hemicelluloses, and lignin. A typical example is acetylated wood, which is produced by reacting acetic anhydride with the wood hydroxyl groups for reducing water absorption and increasing the dimensional stability and biological resistance to fungi and insects.^2^ On the contrary, passive modification of wood involves the filling of lumens as well as the diffusion of chemicals into the cell wall without involving any chemical reaction with the wood polymers. For example, MF-modified wood is produced by impregnation with a prepolymer, followed by a curing step. The changes in wood properties are due to the lumen-filling and polymer-wood cell wall reinforcement by mechanical interlocking.^3,4^ Some types of prepolymer/monomer modification may involve active modification through the covalent bond formation with the wood polymeric constituents, for example, PF-prepolymer or furfuryl alcohol tends to react with the wood components, forming methylene bridges.^5−7^ However, these modified wood products mostly enhanced the biological resistance, durability, and dimensional stability of wood, while the resultant fire resistance generally remained similar to that of unmodified wood.^8−12^

Water-soluble ammonium phosphate salts have been extensively used as fire-retardant (FR) additives both for solid wood and wood-based panels due to their effectiveness, very low toxicity, and competitive prices.^13−16^ The FR mechanism of nitrogen-containing compounds such as ammonium, urea, and melamine primarily involves gas phase dilution resulting from decomposition into non-flammable gases, such as N_2_, at elevated temperatures to decrease the probability of wood contact with O_2_.^17^ Phosphates are very effective in enhancing the fire-retardancy of hydroxyl group-rich polymers, for example, lignocellulosic materials. This is because the phosphates can promote char formation by the dehydration of hydroxyl groups, by creating a protective glassy barrier of polyphosphates, and also by acting as radical scavengers to prevent further heat transfer.^13,18,19^ However, its high leachability in water limits broader applications, as a phosphate salt can eventually be washed away by rainfall and vapor condensation in outdoor conditions, thus reducing the fire retardancy of wood materials with time.^20,21^

The combination of ammonium phosphate salts with a hydrophobic resin has been considered an effective and simple approach to improve the leaching resistance of FR-modified wood, without modifying the formula of the salt. Lin et al. have demonstrated that the water-leaching resistance of guanyl-urea phosphate (GUP)-treated wood can be largely improved by combining it with a water-borne melamine-formaldehyde resin or with poly(furfuryl alcohol).^22−25^ The polymerization of the prepolymer by the formation of a hydrophobic thermoset can possibly entrap the additives. Simultaneously, the passive modification processes of polymer-wood cell wall reinforcement and lumen-filling can provide additional benefits in resin-modified wood, for example, its increased dimensional stability and hardness.^23,25^ In addition, other researchers have reported that water-borne epoxy resin combined with ammonium polyphosphate can improve the fire retardancy, mechanical properties, and the dimensional stability of wood.^26^ This type of strategy can advance the conventional wood-resin modification by upgrading the fire retardancy property. Nonetheless, the modified wood should combine both improved fire resistance and other physical properties; for instance, a small decrease in the dimensional stability of wood may be compensated by the enhancement in fire performance.

It has been noted that the limited research on the active-type FR-wood modification has likely been due to the difficulty of finding economical water-based methods. Ehrhardt et al. have reported that an active-type FR-modified wood can be achieved by the esterification of the wood polymers by organophosphorus compounds.^27^ However, this type of treatment requires the use of an organic solvent, a fact that does not favor scaling-up of the process due to additional solvent re-use requirements.

Herein, a simple new active type of wood modification is demonstrated by applying common, easily obtained, and low-cost reagents, namely, ammonium dihydrogen phosphate (ADP) and urea. These chemicals are mainly limited to interior-use FR-wood products due to their high hygroscopicity. In this work, Scots pine wood (*Pinus sylvestris L.*) was chemically modified by ADP and urea at a temperature of 150 °C, which enabled the hydroxyl groups of wood polymers to graft phosphate and carbamate groups.^28−35^ Further, such modified wood specimens were subjected to water-leaching for 2 weeks, according to European standard EN 84, in order to investigate their potential leaching resistance. In addition, detailed characterization of the modified wood using evolved gas by thermogravimetric analysis coupled with quadrupole mass spectrometry (TGA-QMS), thermal behavior by TGA, chemical functionalities by Fourier transform infrared (FTIR) spectroscopy, X-ray photoelectron spectroscopy (XPS), and solid-state ^13^C and ^31^P magic-angle-spinning (MAS) NMR, morphology and elemental distribution analysis by scanning electron microscopy coupled with energy-dispersive X-ray spectrometry (SEM–EDX), the fire performance by limiting oxygen index (LOI) and cone calorimetry, and mechanical property evaluation by four-point bending tests were conducted.

## Results and Discussion

### Evolved Gas Analysis and Color Changes of Wood during Modification

**Figure 1:**
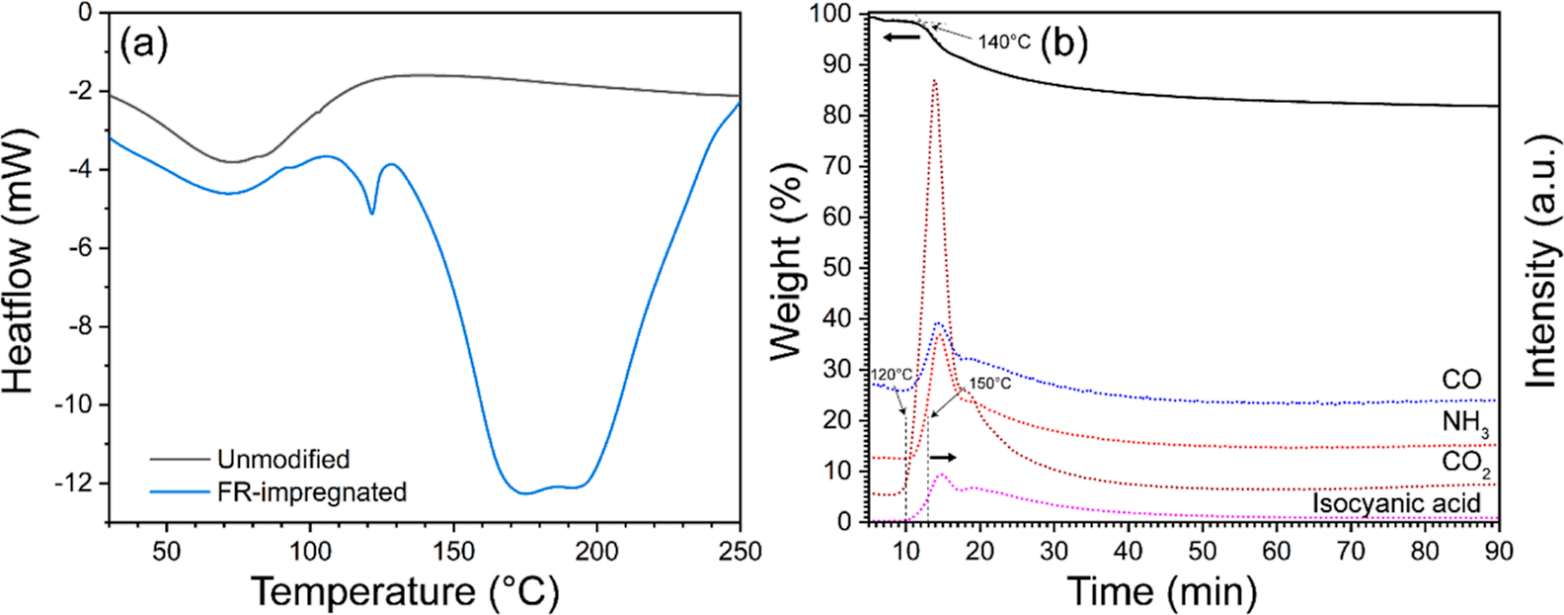
(a) DSC curves of unmodified and FR-impregnated wood, and (b) gas evolution investigated by TGA-QMS of FR-impregnated wood dried at 40 °C. Evolved gases: CO, NH_3_, CO_2_, and isocyanic acid analysis during the present FR modification process.

For the determination of the suitable reaction temperature for modification, unmodified and FR-impregnated wood specimens without heating at 150 °C were subjected to differential scanning calorimetry (DSC) analysis, presented in Figure 1a. The maximum temperature was set to 250 °C because above 250 °C was unrealistic for the thermal treatment as the dramatic deterioration of mechanical properties may take place.^36^ The unmodified wood showed an endothermic broad peak from 30 to 120 °C, with the maximum temperature peak at 80 °C, which is mainly related to the evaporation of the bound water. There are no visible peaks between 120 and 250 °C, although the degradation of polysaccharides starts slowly at around 160 °C. The typical DSC response of wood happens above 300 °C and is typically attributed to the exothermic thermal decomposition of polysaccharides.^37^ On the other hand, the FR-modified wood exhibited two endothermic peaks in the DSC curve: a small peak at 120 °C and a broader peak between 140 and 250 °C with a bimodal distribution at ca. 170–200 °C. The peak at 120 °C is related to the melting of urea.^38^ On an increase of temperature above 140 °C, urea decomposes on NH_3_ and isocyanic acid, the latter readily reacting with the hydroxyl groups of wood polymers, forming carbamate groups. Simultaneously, the melted urea swells into the wood cell walls and acts as a catalyst for the nucleophilic substitution of hydroxyl groups in wood during the phosphorylation.^39,40^ The reaction reached its maximum rate at ca. 170 °C. The bimodal distribution at 170–200 °C was due to the thermal decomposition of ADP into NH_3_ and phosphoric acid at around 200 °C.^41^

The gas evolution of FR-impregnated wood dried at 40 °C was further analyzed by TGA coupled with QMS, as presented in Figure 1b. The analysis can provide real-time information on the gas evolution of the target molecular mass. The emission of isocyanic acid is principally initiated from the decomposition of urea. The highly reactive isocyanic acid can also participate in side reactions, such as its reaction with water, which can result in the evolution of NH_3_ and CO_2_, and its reaction with urea to form a biuret derivative.^38,42^ Thus, the emission of NH_3_ may be attributed to the decomposition of urea and the side reaction of isocyanic acid. The emission of CO and CO_2_ comes from the thermal decomposition of wood polymers.^43^ Additionally, it was noticed that the gas emission of the target molecular mass was reduced after about 20 min at 150 °C without a significant change of the sample weight. Moreover, this was speculated to be the required both reaction time and temperature. Thus, it could be used as an indicator of what the inner part of the wood should reach after the process was scaled up.

After the FR modification process, the color of the specimens changed from its original light yellowish to a dark brown color (see Figure S1a,b in the Supporting Information). The color differences as defined by the CIELAB color system are presented in Figure S1c. The three coordinates L*, a*, and b* represent the lightness (0 to 100), green/red parameter (−60 for green, +60 for red), and blue/yellow parameter (−60 for blue, +60 for yellow), respectively. The FR-modified wood had increased darkness and redness without any significant change in the blue/yellow parameter. The color change of the wood was influenced by the conditions during thermal modification treatment and resulted from an autocatalytic reaction; the cleavage of the O–acetyl bond in hemicellulose while forming acetic acid as well as other organic acids such as formic acid, which can further hydrate the monomeric sugar units into furans such as furfural and 5-hydroxymethylfurfural (HMF).^36^ The acidic ADP/urea solution might also influence the thermal modification. Simultaneously, the formation of unsaturated carbon–carbon bonds, such as the condensation of formed furfuryl structures, including phenolic compounds from lignin degradation, oxidation of polysaccharides, or extractive degradation, can lead to intense chromatic changes in the wood specimens.^36^ The color changes showed a similarity with the color changes of the conventional thermal modification, that is, the wood becomes darker and brownish. Such significant color changes can be applied as a quality control tool in larger-scale production.^44,45^

### Chemical Functionality Identification

**Figure 2:**
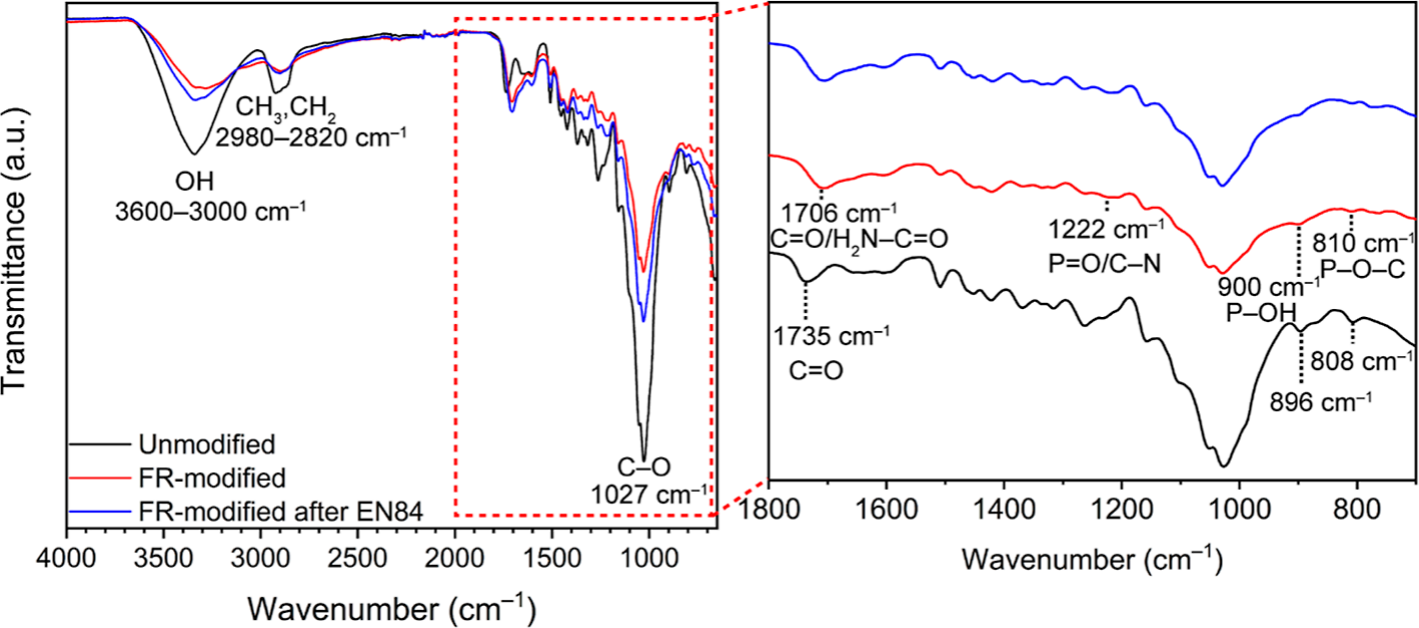
FTIR spectra of the unmodified, FR-modified, and FR-modified wood after the EN84 standard.

The chemical functionalities of the specimens analyzed by FTIR are presented in Figure 2. The bands of the unmodified wood were assigned to the typical wood polymers cellulose, hemicelluloses, and lignin.^23^ The band in the region from 3600 to 3000 cm^–1^ came from the O–H stretching of cellulose, hemicelluloses, and lignin.^46,47^ The band’s intensity was reduced, and it became broader after the FR modification. This was due to the grafting of phosphate and carbamate groups to the hydroxyl groups and due to the thermal treatment.^31,34,48^ The region of bands at 2980–2840 cm^–1^, which were assigned to the C–H asymmetric and symmetric stretching modes of methoxyl, methyl, and methylene groups in wood, became less pronounced after the FR modification. The band at 1735 cm^–1^ was mainly attributed to the carbonyl, C=O stretching of O–acetyl groups in hemicelluloses, unconjugated ketones, carbonyls, and esters.^47,49,50^ This band was shifted to a lower value (1706 cm^–1^) after the FR modification, as a consequence of the thermal degradation during the modification and by the cleavage of the O–acetyl groups in hemicelluloses with the subsequent release of acetic acid.^51^ This band-shifting can also be attributed to the C=O of the grafted carbamate (H_2_N–C=O) groups.^28,52^ A new weak band at 1221 cm^–1^ is assigned to the P=O stretching vibration of the phosphate groups and the C–N stretching of the carbamate groups.^28,53^ The original peaks at 896 and 808 cm^–1^, related to the C–H out-of-plane bending in wood polymers, became broader after the modification, which could be attributed to the P–OH aliphatic bond and P–O–C bond of the grafted phosphate groups and free ADP (Figure 2).^28,31,32,54^ The FR-modified wood after EN84 exhibited the characteristic weak signals from C–N/P=O, P–OH, and P–O–C bonds, which implied a successful phosphorylation and carbamylation of the FR-modified wood with the formation of stable bonds against the water leaching.

**Figure 3:**
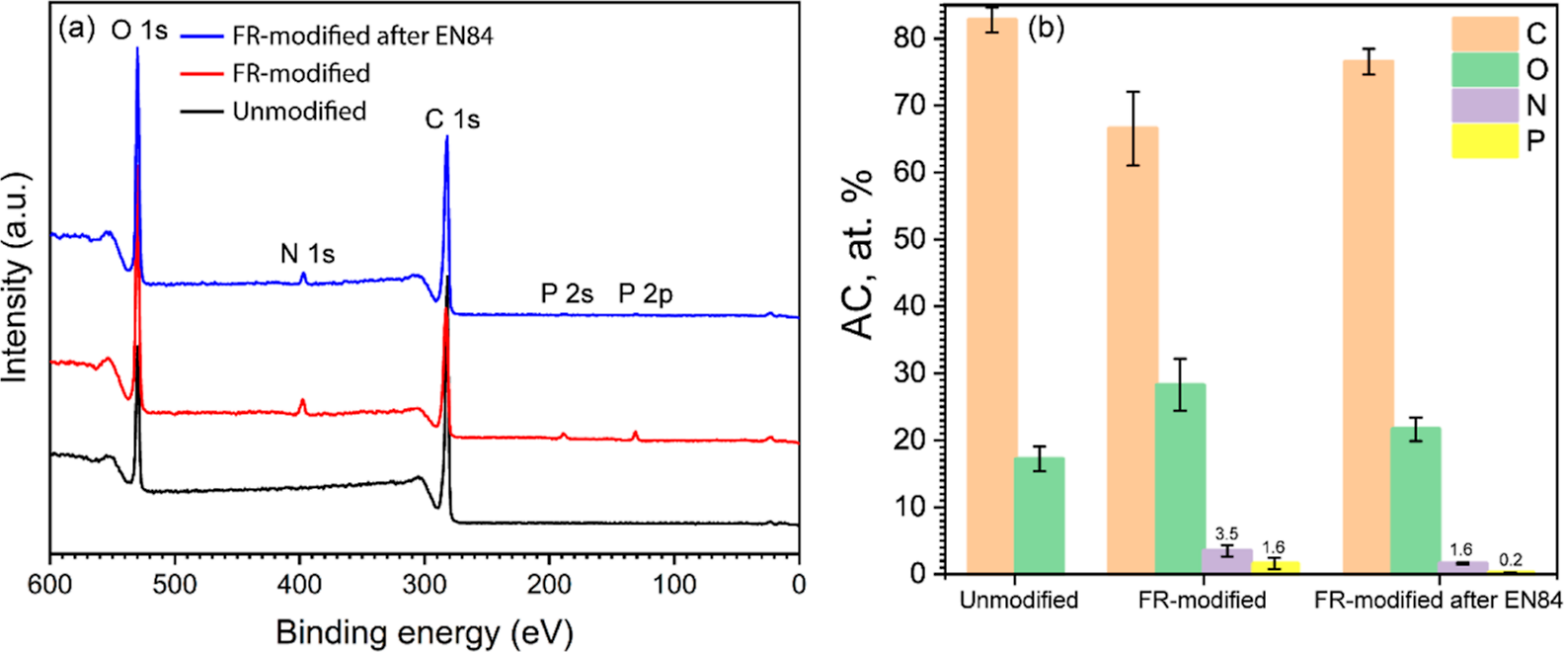
(a) XPS survey spectra and (b) atomic concentrations of C, O, N, and P at the surface of the unmodified wood, the FR-modified wood, and the FR-modified wood after the EN84 leaching test.

X-ray photoelectron spectroscopy (XPS) is a surface analysis technique providing quantitative information on the chemical composition and chemical states of elements within the first 10 atomic layers of material’s surface. XPS survey spectra of the unmodified, FR-modified, and FR-modified wood after EN84 are provided in Figure 3a. The XPS spectra of unmodified wood showed two major peaks at 285 and 531 eV, which corresponded to C 1s and O 1s photoelectron lines, respectively. The FR-modified wood exhibited three additional peaks situated at 400, 189, and 131 eV, which correspond to N 1s, P 2s, and P 2p, respectively.^29,55,56^ In the case of FR-modified wood after EN84, a decreased intensity of these three lines was observed.

The atomic concentrations of C, N, O, and P are shown in Figure 3b, in which the O/C atomic ratio of unmodified wood is about 0.3, that is lower than the reported theoretical O/C ratio of pine sapwood (e.g., 0.5–0.6).^57^ This was most likely because of surface contamination during microtome, that is, a trace amount of lubricant from the microtome blade, as XPS is a surface-sensitive technique, highly sensitive to the outermost 1–10 nm of the material.^58^ The FR modification increased the O/C ratio of wood in view of the fact that the additional oxygen-containing phosphate groups to the wood at the surface, although the wood had been thermally modified, which is understood to reduce the O/C ratio.^48,59^ The decreased O/C ratio, and N 1s and P 2p intensities following the water-leaching test were due to the removal of the unbonded reagents and their derivatives.

**Figure 4:**
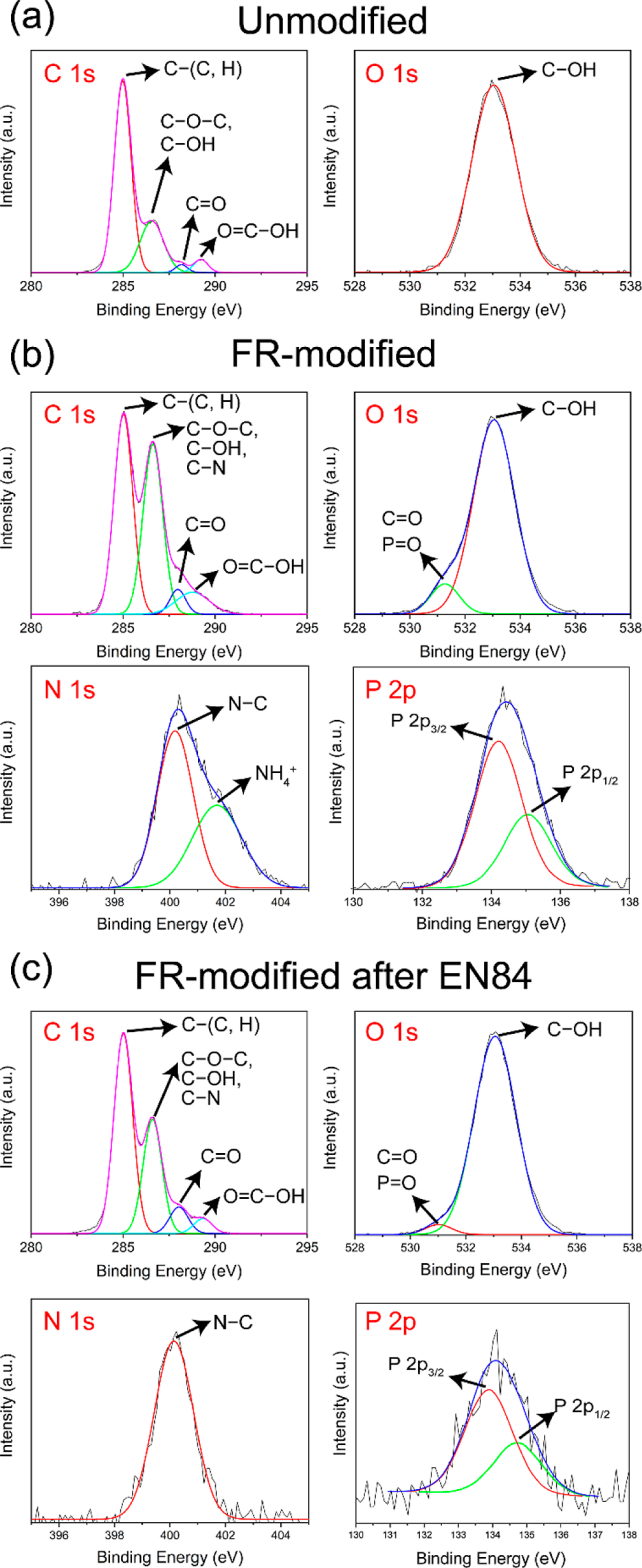
(a) C 1s and O 1s spectra of the unmodified wood, (b) C 1s, O 1s, N 1s, and P 2p spectra of the FR-modified wood, and (c) C 1s, O 1s, N 1s, and P 2p spectra of the FR-modified wood after the EN84 water-leaching test.

The high-resolution XPS spectra of C 1s, O 1s, N 1s, and P 2p are presented in Figure 4a–c). The C 1s spectrum of unmodified wood was fitted by four components at 285.0, 286.6, 287.0, and 289.3 eV, which corresponded to C–(C, H), (C–OH and C–O–C), C=O, and O=C–OH bonds, respectively.^29,31^ In the case of the FR-modified wood, the intensity of the C–(C, H) bonds was relatively reduced probably due to the relative enhancement of the other bonds, that is, the peak centered at 286.6 eV can have an additional contribution from the C–N bonds of the carbamate groups, the dehydration caused by the heat treatment could lead to the formation of keto compounds, for example, furfural and HMF, which can enhance the intensity of C=O bonds, and the evaporation of extractives during the thermal treatment could result in the reduction of the C–(C, H) component.^29,36,60−62^

The O 1s spectrum of unmodified wood showed a peak at 533.0 eV, which was assigned to the C–OH bonds of the wood polymers.^28^ The FR-modified wood showed an additional peak at 531.3 eV, which was assigned to the P=O and C=O bonds of the phosphorylation, carbamylation, and keto group formation during the modification. It is noted that the peak centered at 533.5 eV could also be assigned to the C–O–P bond.^29,31^

The N 1s spectrum of FR-modified wood consists of two components at 400.1 and 401.7 eV, which correspond to C–N bonds, and protonated ammonia (NH_4_^+^) and/or amide as cations, respectively.^60^ The peak at 400.1 eV was assigned to the grafted carbamate groups, as evidenced by its strong existence after the water-leaching test.^53,63^ The peak at 401.7 eV was assigned to the NH_4_^+^ of ADP and the protonated carbamate groups, which were much less visible after the leaching test due to the replacement of NH_4_^+^ by H^+^ and deprotonation.^28,60^

The P 2p spectrum of the FR-modified wood is a doublet (due to spin–orbital splitting) at 134.2 eV (P 2p_3/2_) and 134.9 eV (P 2p_1/2_) assigned to phosphate groups.^28,31,55^ The existence of phosphate groups after the water-leaching test was presumably due to the bonding formation with wood polymers. (See the following discussion of ^31^P MAS NMR data.).

**Figure 5:**
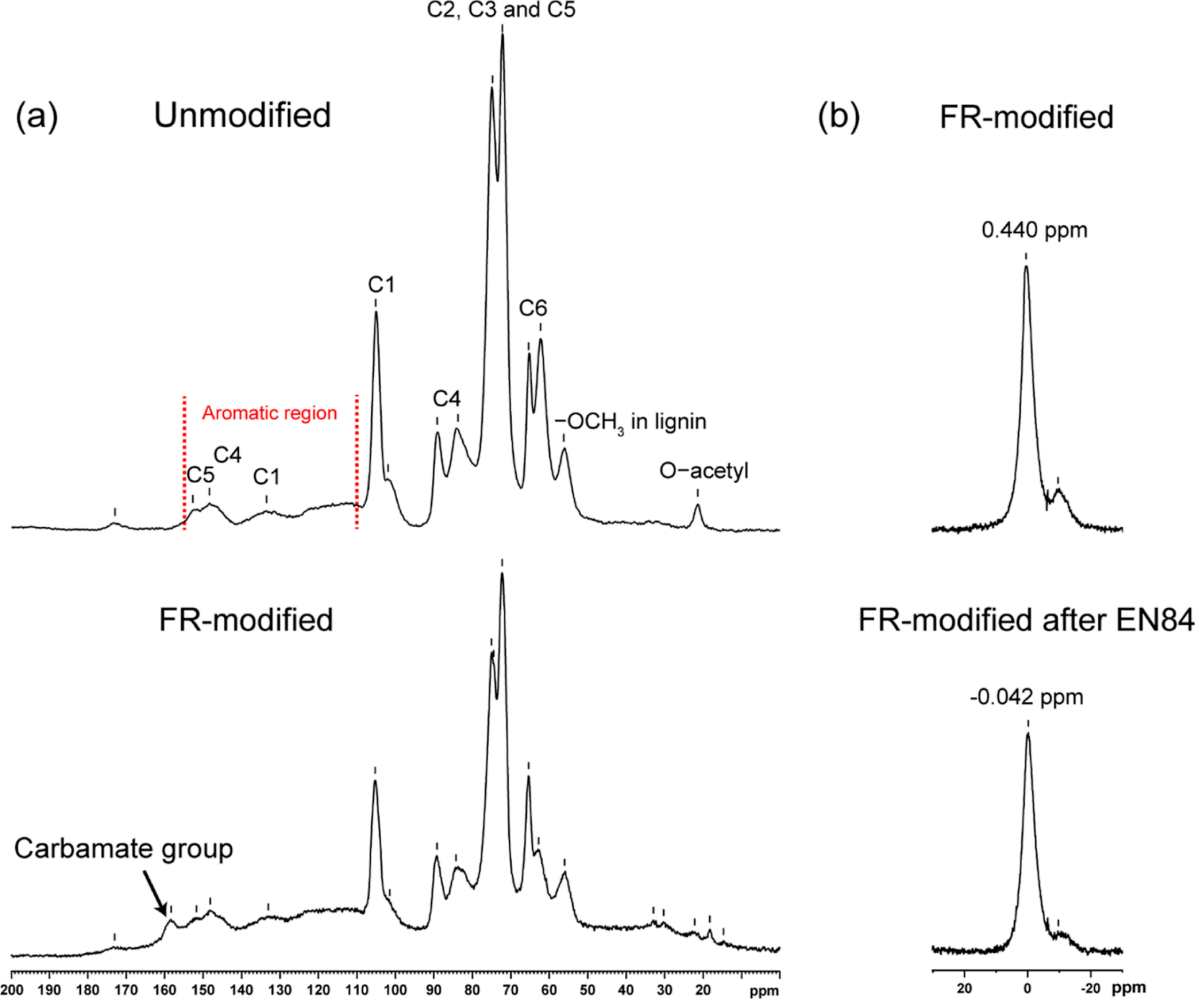
(a) ^13^C CP-MAS and (b) direct excitation ^31^P MAS NMR spectra of the unmodified wood, the FR-modified wood, and the FR-modified wood after EN84. The MAS was 8 kHz.

The natural abundance (1.08 at % ^13^C) ^13^C CP-MAS NMR spectra of the unmodified and FR-modified wood are shown in Figure 5a, while the main chemical shift assignment is listed in Table 1. The study was carried out to confirm the grafting of phosphate and carbamate groups. The isotropic chemical shifts of unmodified wood are assigned to carbon sites in the wood polymers cellulose, hemicelluloses, and lignin. The ^13^C chemical shifts of >C(H)–O–, >C(H)–OH, and −O–CH_3_ carbon sites of cellulose and hemicelluloses are mainly located within the region of 60–105 ppm. The carbon sites in lignin have chemical shifts mainly in three different regions in the ^13^C NMR spectra: aromatic units at 110–155 ppm, β and α carbons at 72–84 ppm, and the methoxy groups at 56 ppm.^64,65^

**Table 1:** Assignment of Main ^13^C Chemical Shifts in the CP-MAS NMR Spectra of Wood

chemical shifts (ppm)	assignment
173	C=O of lignin and carboxyl groups of hemicelluloses^70^
158	C=O of carbamate groups^52,68^
153	C5 of aromatic ring carbons of lignin^70^
148	C4 of aromatic ring carbons of lignin^70^
134	C1 of aromatic ring carbons of lignin^70^
105	C1 of cellulose^71^
102	shoulder of C1 of hemicelluloses^70^
89	C4 of crystalline cellulose^71^
84	C4 of amorphous cellulose;^71^ Cβ of the β–O–4 bond in lignin^72^
75–72	overlapped signals of C2, C3, and C5 cellulose;^71^ Cα of the α–O–4 bond in lignin^72^
65	C6 of crystalline cellulose^71^
62	C6 of amorphous cellulose and hemicelluloses^70,71^
56	methoxy carbon (–OCH_3_) of lignin^64^
21	acetate group in hemicelluloses^70^

The change in the ^13^C NMR spectrum after the FR modification of wood can be clearly observed in the region of C4 and C6 chemical sites of cellulose. The resonance line at 84 ppm assigned to the C4 sites in amorphous cellulose and hemicelluloses has a lower integral intensity compared to that of the C4 sites in the crystalline cellulose (the resonance line at 89 ppm). This is an indication of increased crystallinity in the FR-modified wood compared to the unmodified wood, caused by thermal degradation and a cleavage of glycosidic linkages that preferably started within the less ordered molecules.^66^ As the resonance line at 84 ppm is overlapped with Cβ in lignin, the cleavage of β–O–4 during the heat treatment could also be accounted for in the reduction of the integral intensity of this resonance line.^48^ The thermal degradation is also reflected in a significant reduction in the integral intensity of the resonance line peak at 62 ppm assigned to C6 carbon sites of amorphous cellulose and hemicelluloses. Additionally, the grafted phosphate and carbamate groups could also contribute to the reduction in the integral intensity of C6 resonances.^67,68^ The thermal degradation of hemicelluloses during the FR modification is also supported by the cleavage of the O–acetyl group (at 21 ppm), glycosidic linkages of C1 and C4 sites in hemicelluloses (at 102 ppm), and carboxyl groups (at 173 ppm). The FR modification caused only minor changes for carbon sites in the region from 75 to 72 ppm, which were assigned to C2, C3, and C5 sites of cellulose as well as Cα of lignin. The observation of additional splitting in some of the resonance lines is probably caused by the grafted carbamate and phosphate groups.^29,31,68^ It was also found that the FR-modified wood has additional carbon sites at 158 ppm assigned here to the grafted carbamate groups on wood polymers.^52,68^ This resonance line is different from the original ^13^C=O isotropic chemical shift of urea at 163 ppm.^52^

**Figure 6:**
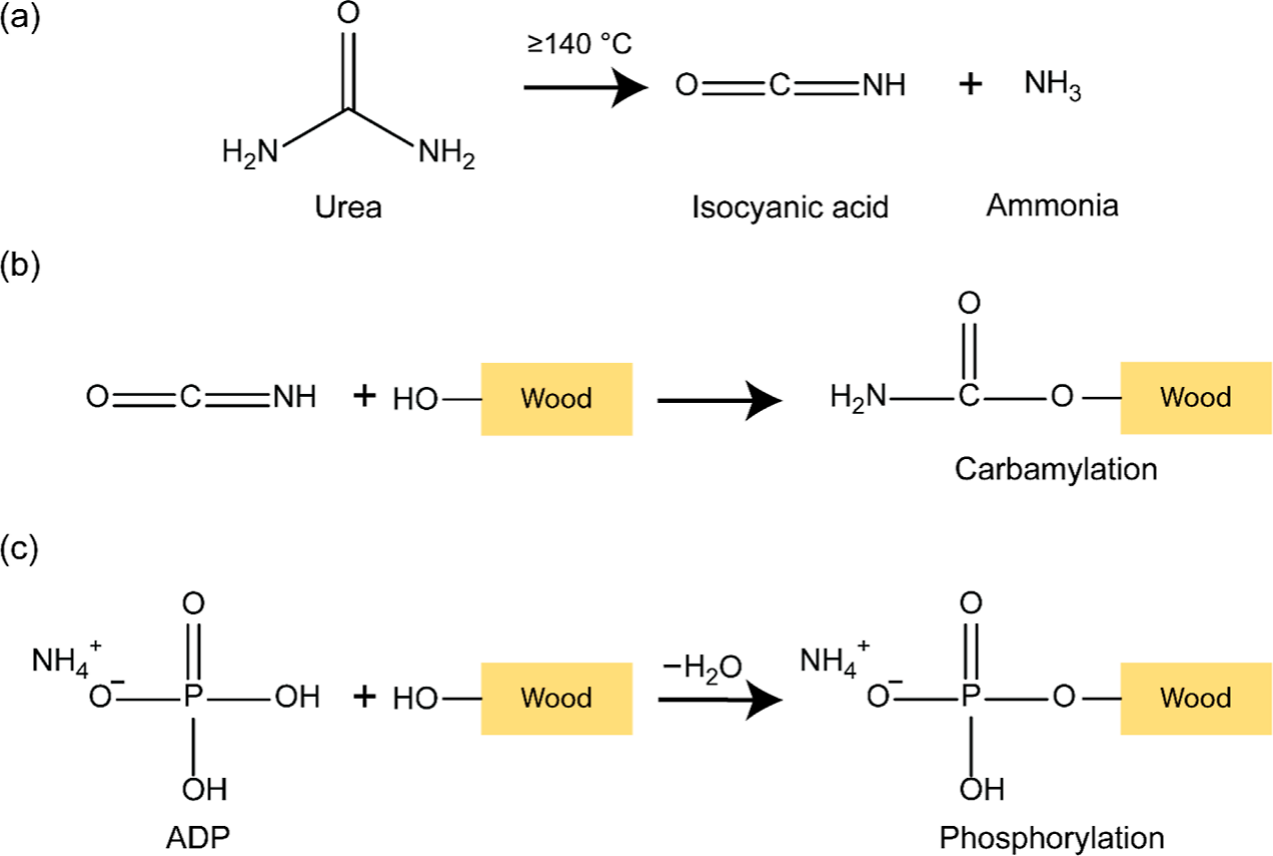
Proposed mechanisms of (a) thermal decomposition of urea into isocyanic acid and ammonia, (b) carbamylation, and (c) phosphorylation of hydroxyl groups of wood polymers.

The direct excitation ^31^P MAS NMR spectra of the FR-modified wood and those after the water-leaching test are shown in Figure 5b. The FR-modified wood has revealed two resonance lines at ca. 0.5 and −10 ppm, respectively. The high-intensity resonance line at +0.44 ppm is assigned to the phosphate group that forms a C–O–P bond with wood polymers as well as the unreacted free ADP.^33^ 85% phosphoric acid was used as the external reference (0.0 ppm). Therefore, a slight deshielding of phosphorus sites was attributed to the effects of alkali NH_4_^+^ cations, which are present in unreacted ADP. The broader and weaker resonance line at −10 ppm was putatively assigned here to the pyrophosphate and polyphosphate groups, which are products of side reactions of phosphate dehydration with the formation of the condensed structure during the FR modification.^32,33,69^ For the FR-modified wood after EN84, the high-intensity resonance line was slightly shifted upfield to −0.04 ppm, probably due to the replacement of NH_4_^+^ by H^+^ during the water-leaching test. The removal of NH_4_^+^ was consistent with the XPS result for N 1s. The reason for the resonance line at ca. −10 ppm becoming less visible was probably because the condensed ADP was mostly homopolymerized without grafting to the wood polymers; thus, it was washed away during the water-leaching EN84 test. We conclude that systematic chemical analysis of FTIR, XPS, ^13^C CP-MAS, and ^31^P MAS NMR unequivocally confirmed the grafting of phosphate and carbamate groups onto the wood polymers, which were not hydrolyzed during the EN84 test. The proposed mechanism of grafting carbamate and phosphate groups on wood polymers is presented in Figure 6a–c.

### Morphology and Elemental Composition

**Figure 7:**
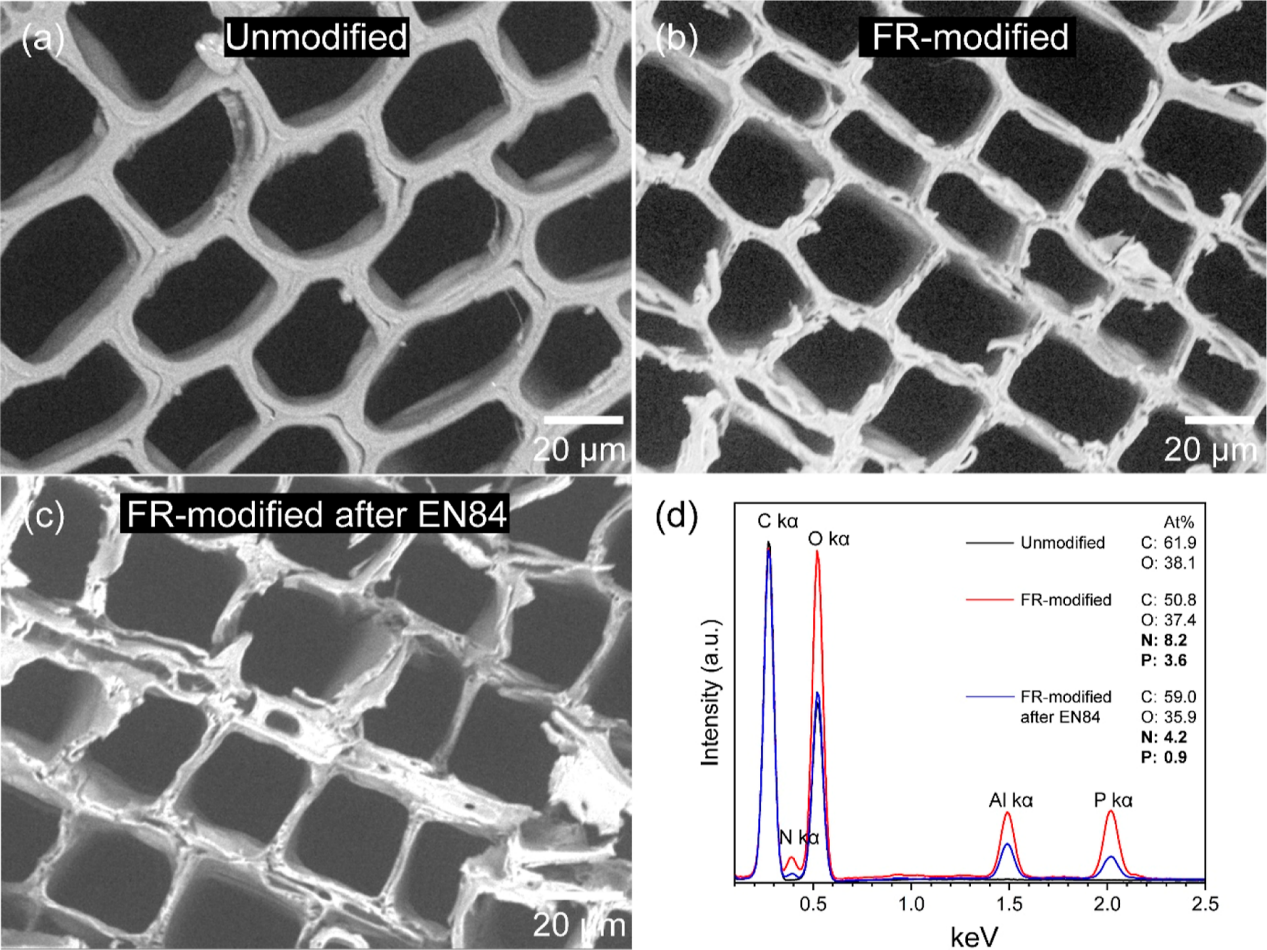
SEM images of (a) the unmodified wood, (b) the FR-modified wood, and (c) the FR-modified wood after EN84, and their corresponded (d) EDX spectra.

The morphology of wood in the axial direction (i.e., cross sections) was observed by scanning electron microscopy (SEM) and is presented in detail in Figure 7a–c. In unmodified wood, the cell walls are separated by hollow cavities, that is, lumens. The lumens, axial resin canals, and tracheids are the major paths for liquid transportation along the axis of the living tree stem. Liquids can also be transferred along the horizonal direction by ray cells and pits. Nanopores in the cell wall allow the diffusion of small molecules. These microstructure features of the wood are the main aspects for active modification, as chemical compounds need to pass through the lumens and diffuse into the cell wall for reacting with the wood polymers.^73^ The liquid uptake of solutions and diffusion into the cell wall depend on various parameters, for example, wood anatomical features, moisture content, viscosity of the solution, impregnation procedure, and the molecular size of chemicals. European pine sapwood is commonly chosen for wood modification processes because of the presence of window-like cross-border pits between the ray parenchyma and longitudinal tracheid, which allow a high permeability of impregnation solutions. Optimizing the moisture content of wood can lead to more effective penetration.^74^ Solutions that do not contain prepolymers, for example, an ADP/urea water solution, achieve a better penetration due to their low viscosity and molecular size. The impregnation procedure, such as applying vacuum before pressure impregnation, can remove entrapped air and result in a higher loading of chemicals into the wood structure.^1^

The FR-modified wood appeared to have a similar morphology as that of the unmodified wood, Figure 7b. However, images showed more loose fibers on the cell wall. This was because the hydrothermal acidic conditions during the modification caused the degradation of hemicelluloses, yielding acetic acid and sugars, which led to a stiffening of the cell wall and the material became highly sensitive to cutting with a blade during sample preparation.^51,62,75^ The water-leached FR-modified wood had further damage, as depicted in Figure 7c. This is potentially attributed to the degradation of the cell wall caused by acidic water (see Table S1 in the Supporting Information) during the lengthy leaching test.^75^ The acidic character of water was from the degradation byproducts such as acetic acid and formic acid, as well as from free leached ADP and its condensed byproducts.

The elemental composition of wood specimens analyzed by SEM–EDX is presented in Figure 7d. The EDX spectrum of unmodified wood was primarily composed of elemental C and O, which were mainly attributed to the wood polymers cellulose, hemicelluloses, and lignin.^23^ In the case of the FR-modified wood, the elemental N and P were characteristically detected as a result of the introduction of ADP and urea during impregnation of the wood specimens. Note that the elemental Al came from the sample holder. The FR-modified wood after EN84 showed a reduction in the elemental P and N because of the extraction of unreacted reagents and their derivatives. The O/C ratios of the unmodified, the FR-modified, and the FR-modified wood after EN84 were found to be 0.61, 0.74, and 0.61, respectively. The O/C ratio of the unmodified wood is close to the expected theoretical value of 0.5–0.6.^57^ The technique of EDX is less surface-sensitive than XPS, operating to a depth of several μm of the surface.^76^ Additionally, the increase of O/C ratio after the FR modification was due to the introduction of phosphate groups into the wood structure. This O/C ratio was reduced after the water-leaching due to the removal of free and condensed ADP. Regarding the elemental distribution of each element, EDX mapping is presented in Figures S2 and S3 (see the Supporting Information), showing that the distribution of the elemental N and P overlapped with the elemental C and O. Such results indicated that ADP/urea diffused into the cell wall following with the phosphorylation and carbamylation of the hydroxyl groups during the thermal treatment.

### Thermal Behavior

**Figure 8:**
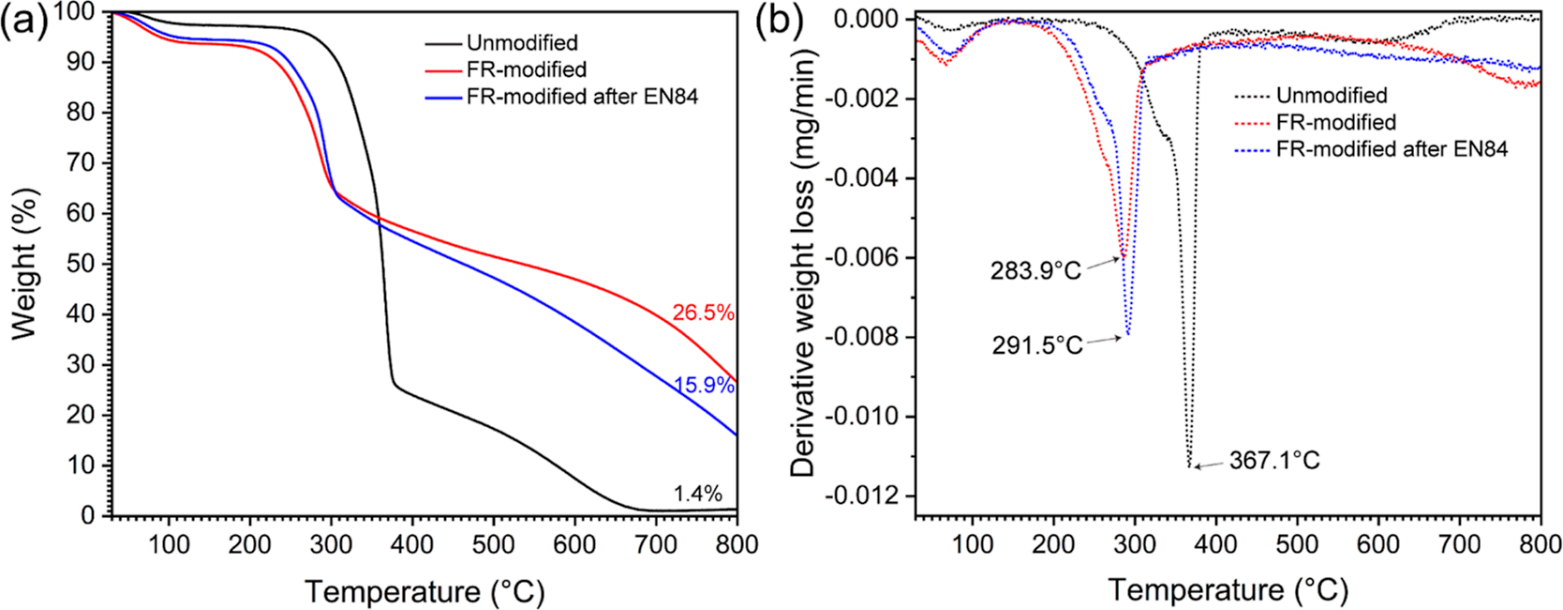
(a) TGA and (b) DTG curves of the unmodified wood, the FR-modified wood, and the FR-modified wood after EN84.

The thermal behavior of wood specimens evaluated by the thermogravimetric analysis (TGA) with its first-order derivative thermogravimetric (DTG) is presented in Figure 8a,b. The TGA curve of the unmodified wood displayed three stages of mass loss. The first stage, from 30 to 110 °C, was assigned to the evaporation of the bound water within the wood matrix. The second stage, from 250 to 380 °C, with the maximum decomposition temperature at 367.1 °C, was assigned to the rapid thermal decomposition of hemicelluloses and cellulose by cleaving the glycosidic linkages in polyoses, O–acetyl bonds, decarboxylation, decarbonylation, dehydration, and also the production of volatile degradation products such as levoglucosan, furfural, acetic acid, CO_2_, CO, and H_2_O.^77−79^ The third stage, from 380 to 650 °C, was mainly due to the thermal degradation of lignin by cleavages between monolignols and the vaporization of monomeric phenols. The weight loss above 650 °C was closely related to the decomposition of carbonized and aromatic structures.^23^ Finally, the remaining residue at 800 °C was 1.4% of the initial mass.

In general, the FR modification strongly influenced the thermal behavior of the wood. The main decomposition temperature was shifted to a lower temperature at 291.5 °C, which was associated with the thermal degradation path that was altered by free ADP and grafted phosphate groups. Dehydration became favored with the promotion of char residues at elevated temperatures.^80^ The dehydration of the phosphate groups can also lead to the formation of condensed polyphosphates, which may act as protective barriers.^18^ Urea can synergistically catalyze the decomposition of lignocellulosic material caused by the phosphate groups at a low temperature and thus promote the formation of char residues, although the grafted carbamate group might not be attributed to the charring promotion.^81,82^ Therefore, up to 26.5% of the initial mass at 800 °C was observed as char residue. Since the water-leaching test led to a partial loss of FR additives from wood, less char residue was noted at 800 °C (15.9%), and a lower degree of the main decomposition temperature shifting was observed in the TGA curve of the FR-modified wood after water-leaching.

### Fire Performance

**Figure 9:**
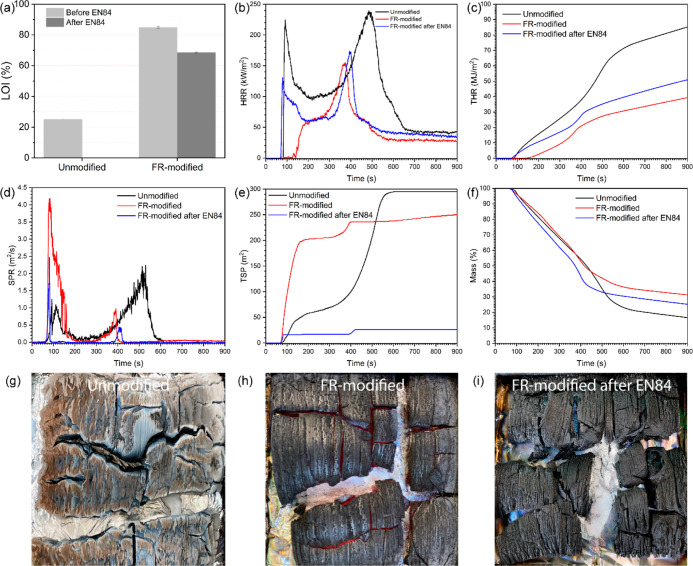
(a) LOI result, (b–f) cone calorimetry test of heat release rate (HRR), total heat release (THR), smoke production rate (SPR), total smoke production (TSP), and mass loss of the unmodified wood, the FR-modified wood, and the FR-modified wood after EN84, and (g–i) the color difference of the char residues after the test.

The fire performance of unmodified and FR-modified wood examined by the limiting oxygen index (LOI) is presented in Figure 9a. Typically, the LOI is used to examine the minimum O_2_ concentration in the N_2_/O_2_ mixture that is required to support the combustion. This clearly indicated a higher fire resistance of the tested material, only when the value was over the threshold of 50%.^83^ Results in this work proved that the incorporation of ADP and urea significantly improved the fire performance of wood due to the incorporation of the phosphate groups. The grafted carbamate groups probably did not contribute to an improved fire retardancy by themselves, as the polymerized carbamate (polyurethane) usually has a similar LOI as that of the unmodified wood.^84^ Herein, the FR-modified wood even after EN84 exhibited a superior LOI value, nearly 70%. To note, this LOI was significantly higher than the previously reported for fire-retardant modified wood.^23,24,85^ In order to verify this result, a cone calorimetry test according to ISO 5660-1 was carried out on the wood specimens.

Generally, the cone calorimetry test evaluates critical fire performance parameters such as the heat release rate (HRR), total heat release (THR), smoke production rate (SPR), total smoke production (TSP), mass loss, and time to ignition (TTI). Furthermore, a reaction-to-fire classification prediction is also possible based upon the parameters. The results for the unmodified, the FR-modified, and the FR-modified after EN84 wood are shown in Figure 9b–f and tabulated in Table 2. The HRR in Figure 9b of the unmodified wood exhibits two peaks. The first peak (pHRR_1_) is related to the oxidation of the wood surface with the release of heat. The dip or decrease in HRR was because of the surface charring, which in turn lowered the heat release. Upon continual exposure of the surface of the char to irradiation, the char broke with increasing surface porosity, which allowed air penetration further into the inner part of the wood and the combustion started again. This behavior was observed as a second peak of HRR (pHRR_2_) in Figure 9b. The following HRR reduction was due to the flameless burning (glowing) of the char after the volatile products were consumed.^23,86^

**Table 2:** Time to Ignition (TTI), Fire Growth Rate (FIGRA), Maximum Average Rate of Heat Emission (MARHE), Total Heat Release (THR), Peak Heat Release Rate (pHRR_1_ and pHRR_2_), Total Smoke Production (TSP), and Fire Class Prediction of the Unmodified, FR-Modified, and FR-Modified Wood after EN84

	unmodified	FR-modified	FR-modified after EN84
TTI (s)	24 ± 6	147 ± 10	19 ± 3
FIGRA (kW/m^2^s)	0.6 ± 0.0	0.4 ± 0.1	0.5 ± 0.1
MARHE (kW/m^2^)	143 ± 6	58 ± 4	92 ± 14
THR (MJ/m^2^)	85 ± 1	39 ± 2	55 ± 6
pHRR_1_ (kW/m^2^)	215 ± 8	65 ± 6	140 ± 11
pHRR_2_ (kW/m^2^)	225 ± 14	151 ± 20	164 ± 24
TSP (m^2^)	372 ± 33	267 ± 9	24 ± 7
fire class prediction	**D**	**B**	**B**

In respect of the FR-modified wood, pHRR_1_ was largely reduced due to the char layers building up progressively in the ignition stage. With continuing heat generated on the wood surface, the fire started propagating with an increasing heat release, shown as pHRR_2_. It was noticed that pHRR_2_ was much lower than that of the unmodified wood because of the FR modification. The introduction of FR additives (ADP/urea) was active in both the condensed and vapor phases, each reducing the fire hazard. The condensed phase mode of action was by promoting the char formation through dehydration of the polymeric structure. The formation of carbonaceous char can certainly reduce the release of volatiles. Additionally, the formation of polyphosphate can behave as multiple protective insulation layers, and the produced radical P^•^ can act as a radical scavenger to interrupt the chain reaction of combustion by reacting with free radicals H^•^ and O^•^. The gas-phase mode of action is by releasing water vapor and non-combustible gases to reduce the combustion efficiency and/or to quench flames.^13,18,80^ In addition, the FR-modified wood after EN84 exhibited a suppression of HRR and THR due to the presence of FR additives into the wood matrix.

Apart from the heat release, the production of smoke is also critical for measuring the fire hazard properties of the material. The FR-modified wood promoted the smoke production rate (SPR) at the ignition stage, although the total smoke production (TSP) was rather reduced. The promotion of smoke production is caused by the combustion of the generated degradation byproducts during the thermal treatment,^48,87^ although FR additives ADP/urea can partially reduce the smoke production.^83^ The smoke suppression of ADP/urea is limited since conventional systems comprising ammonium phosphate-based FRs are typically combined with inorganic compounds like boric acid, disodium tetraborate, and aluminum or magnesium oxides.^83,88^ Interestingly, FR-modified wood after EN84 showed a significant reduction of the SPR and TSP parameters. This occurred due to the extraction of smoke production-related compounds, for example, furfural and HMF, during the water-leaching test.^36^

The mass loss and mass of the final residue are frequently used for evaluating the thermal stability of materials.^86^ The FR modification of wood significantly reduced the mass loss after pHRR_2_ due to the large amount of thermally stable char formed during the combustion. This fact is closely related to the color difference of the char residues after the test, where the FR-modified wood was much darker than the unmodified wood, Figure 9g–i. The result is consistent with the TGA.

The calculated fire growth rate (FIGRA), maximum average rate of heat emission (MARHE), and the prediction of the reaction-to-fire classification are tabulated in Table 2. The FIGRA value is used to estimate the flashover time of the fire. In general, a lower FIGRA indicates a better fire performance. In addition, the MARHE parameter is originally described in the European standard EN45545-2:2013 and used as a criterion for comparing the fire hazards of different combustible materials.^89^ A lower MARHE implies a better fire performance. The results in Table 2 demonstrate that both FIGRA and MARHE were significantly reduced for the FR-modified wood. The FR-modified wood after EN84 has slightly increased values of FIGRA/MARHE due to the removal of free ADP/urea.

The prediction of the reaction-to-fire classification is presented in Table 2. The classification is originally described in European standard EN 13501-1, based on the medium-scale test of the single burning item (SBI).^90^ The classification divides the property into 6 classes, from high to low levels A, B, C, D, E, and F, respectively. The best fire classification for the combustible products, for example, wood, can reach is B.^91^ The typical wood without FR treatment is D. As the SBI test is relatively time-consuming and expensive, the fire classification prediction based on the bench-scale cone calorimeter test is frequently used before further scale-up.^92^Table 2 shows that both the FR-modified wood and that after water-leaching fulfilled the fire classification B. The result indicates that the FR modification has a high potential for the further development of exterior-use fire-retardant timbers. Overall, the fire hazard tests of LOI and cone calorimetry affirmed the improvement of the fire-retardancy even after the severe water-leaching test EN84.

### Physical and Mechanical Properties

Weight percentage gain (WPG) presents the loading amount of chemicals within the wood matrix after the treatment and is shown in Table 3. As the FR modification with ADP/urea also involved the emission of small molecules and a thermal degradation, this led to the partial loss of WPG. The FR-modified wood increased by 14.8% of its initial weight after the modification. The water-leaching test EN84 shows the loss of WPG due to the removal of the water-soluble wood degradation byproducts, unreacted reagents, and their derivatives.

**Table 3:** WPG, WPG Loss after EN84, EMC, and BC of the FR-Modified Wood

WPG (%)	WPG loss after EN84 (%)	BC (%)	EMC before EN84 (%)	EMC after EN84 (%)
14.8 ± 0.6	–7.8 ± 0.6	3.3 ± 0.7	10.8 ± 0.5	12.4 ± 0.2

The bulking coefficient (BC) was calculated by comparing the macroscopic dimensions before and after the treatment. The macroscopic volume bulking was mainly because the chemicals diffused into cell walls while occupying the nanopores during the chemical modification process. In contrast, the thermal modification tended to shrink the volume caused by the degradation of wood components.^48^ The positive BC value shown in Table 3 was because the bulking of the non-grafted and grafted phosphate and carbamate groups was more significant than the shrinkage caused by the thermal modification. The result was in coherence with the elemental mapping by SEM–EDX, in that elemental N and P from ADP/urea were found within the cell wall.

The hygroscopic wood absorbs moisture from the atmosphere. The steady state of MC at a certain RH and temperature is defined as equilibrium moisture content (EMC). The EMC can reflect the hydrophilicity, which could influence the bio-resistance and the dimensional stability of the wood specimens.^93^ The FR-modified wood showed a reduction of EMC (Table 3). This was attributed to the irreversible thermal modification by reducing the total amount of accessible OH groups in hemicellulose and amorphous cellulose.^13,48,94^ Additionally, the grafted carbamate group could also reduce the moisture uptake.^95^ On the other hand, the hydrophilicity of the grafted phosphate group, free/condensed ADP, and urea’s derivatives could increase the moisture absorption. Consequently, the EMC of the FR-modified wood was higher than that of the conventional thermally modified wood, about 7%.^36,43^ The FR-modified wood after EN84 shows higher EMC, which is attributed to the reversible reaction of the annealing of amorphous wood polymers as well as the removal of the accumulated degradation products.^96,97^ In the case of reversible reactions, the amorphous wood polymers annealed and realigned during the thermal modification process are recovered to their initial conformation after soaking in water. Therefore, the capacity of the moisture uptake is increased. In the case of the removal of the accumulated degradation products, the accumulation of the thermal degradation products that occupies the nanopores is removed during the water soaking, and the void can absorb moisture.

**Figure 10:**
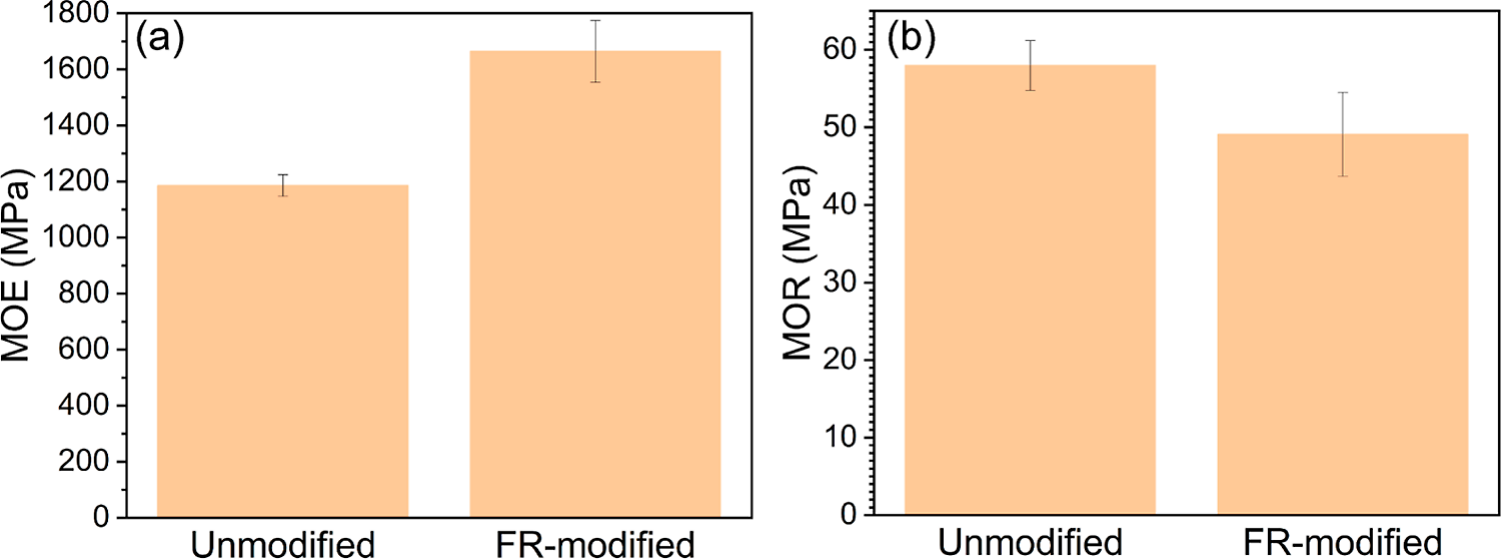
(a) Modulus of elasticity (MOE) and (b) modulus of rupture (MOR) of the unmodified and FR-modified wood.

The modulus of elasticity (MOE) and modulus of rupture (MOR) of the specimens were evaluated by a four-point bending test through loading the specimen with an increasing force until breaking occured. The FR modification increased 40% of MOE and did not significantly decrease the MOR of the wood specimens (Figure 10). The wood specimens became stiffer due to the thermal degradation of hemicelluloses with a relatively increased amount of crystalline cellulose during the thermal modification.^98,99^ The result supported the enhancement of crystallinity observed by ^13^C CP-MAS NMR. Additionally, the amorphous cellulose in a moist high-temperature environment could also reorient, giving rise to an increase in the crystallinity of cellulose,^100^ and similar mechanical properties have been reported for conventional thermally modified wood.^43^ This was probably because urea acted as a buffer to alleviate the potential severe mechanical property deterioration during the FR modification caused by a solely ADP treatment.^101^

## Conclusions

A protocol for the active modification of solid wood using a green and facile method of impregnation of wood with an ADP/urea solution followed by a thermal treatment was successfully demonstrated, showing a high fire-retardancy and a good water-leaching resistance of the processed wood. We found that the phosphate and carbamate groups from the ADP/urea were grafted to the hydroxyl groups of wood polymers, as confirmed by combined results from XPS, FTIR, and solid-state ^13^C and ^31^P NMR. During the FR modification process, the formation of isocyanic acid due to the thermal decomposition of urea is proposed to be the mechanism for further grafting the carbamate groups within the cell walls. Additionally, due to the acidic ADP/urea solution and high-temperature process conditions, a thermal degradation of the amorphous cellulose and hemicelluloses also occurred. This was also reflected in the color change from yellowish to brownish color of the wood. The conditioned modified wood had an excellent fire performance even after the severe water-leaching test. This was proved by the reduction in the heat release rate and the promotion of char residues during the cone colorimeter test. Meanwhile, the smoke production was not significantly suppressed, although the smoke production was significantly reduced after the water-leaching test. The predicted reaction-to-fire reached the highest classification for wooden materials, even after the water-leaching test. The modified wood was slightly stiffened without dramatically losing its bending strength. Overall, the new type of high-performance FR-modified wood is reported and has the potential for further optimization and adoption of FR-protocols in industrial thermal modification processes.

## Experimental Section

### Materials

Knot- and crack-free Scots pine (*P. sylvestris L.*) sapwood was obtained from a sawmill in Northern Sweden. Ammonium dihydrogen phosphate (ADP, H_6_NO_4_P, 98% purity) was obtained from Alfa Aesar (Massachusetts, USA), and analytical-grade urea (CH_4_N_2_O) was purchased from VWR (Stockholm, Sweden). Deionized (DI) water was obtained from a Milli-Q Direct 8 water purification system of Merck KGaA (Darmstadt, Germany). All chemicals were used as received without any purification.

### Wood Modification Procedure

Twenty wood specimens of dimension 10 × 10 × 150 mm and twenty specimens of dimension 100 × 10 × 100 mm in tangential (*T*) × radial (*R*) × longitudinal (*L*) directions, respectively, were conditioned at 20 °C and 65% relative humidity (RH) to reach an equilibrium moisture content (EMC) of approximately 12.5%, before measuring the mass (*m*_1_) and volume (*v*_1_). The density of the conditioned wood specimens was 500 ± 50 kg/m^3^.

Deionized water (DI) solution containing 14.4 wt % of urea and 7.2 wt % of ADP (i.e., the urea/ADP mole ratio was about 4:1) was prepared for the impregnation of wood. The pH of the solution was 4.3. The impregnation procedure was accomplished by immersing the specimens in the aqueous solution before applying 30 min of vacuum at 20 mbar, and afterward, 15 bar pressure for 1 h. The excess liquid on the surfaces was removed by tissue paper before drying at 40 °C for 24 h and at 150 °C for 24 h. After that, the mass (*m*_2_) of the wood specimens was measured. The specimens were then placed in a laboratory conditioning chamber at 20 °C and 65% RH, and their mass (*m*_3_) and volume (*v*_2_) were recorded before further analyses. The weight percentage gain (WPG), bulking coefficient (BC), and EMC were calculated according to 1, respectively.
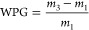
1
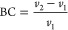
2
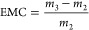
3

### Accelerated Aging Test

Five wood replicates, from each dimension, were subjected to the accelerated aging test according to European standard EN 84:2020^102^ The specimens were fully immersed in a fivefold volumetric excess of DI water before applying 20 min of vacuum in a desiccator. The water was subsequently changed 10 times within a 14 day leaching period. The first water change took place 2 h after the vacuum, while the remaining nine water changes were carried out within a period of no less than 1 day and no more than 3 days. The wet specimens were then conditioned at 20 °C and 65% RH until the mass was constant and then recorded (*m*_4_). The WPG after EN84 was calculated by eq 4, which is used to calculate the WPG loss during EN84 by eq 5.
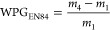
4
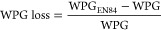
5

### Characterization

For evolved gas analysis during the FR modification, thermogravimetric analysis coupled with quadrupole mass spectrometry (TGA-QMS, Netzsch STA 449 F3 Jupiter coupled with TGA/STA QMS 403 D Aëolos, Selb, Germany) was performed from 50 to 150 °C at a heating rate of 10 °C min^–1^, following an isothermal step at 150 °C for 80 min under an argon flow rate of 50 mL min^–1^. Each specimen was oven-dried at 40 °C to reach a constant mass before analysis. Differential scanning calorimetry (DSC, Mettler Toledo DSC 821e, Columbus, OH, USA) was carried out at a heating rate of 10 °C min^–1^ from 30 to 250 °C under a N_2_ flow rate of 80 mL min^–1^ using three replicates from the specimen. Surface material from the specimen, measuring 4 ± 1 mg, was collected by a microtome steel blade (Leica DB80 LX, Nussloch, Germany) and then enclosed in a standard aluminum pan (Mettler Toledo, Columbus, OH, USA) with a pierced lid. An empty aluminum pan was used as a reference. The evaluation was made via STARe Evaluation V16.20 software (Mettler Toledo, Columbus, OH, USA). The color of the specimens was measured on freshly planned surfaces (planning depth ∼ 0.5 mm, belt sander, sandpaper grit size 120) in six replicates using a Minolta Chroma Meter CR-410 (Konica Minolta Inc., Osaka, Japan) on a measurement area having a diameter of 55 mm. Fourier transform infrared spectroscopy (FTIR), using a Perkin Elmer FTIR Frontier spectrometer (Waltham, MA, USA) equipped with a UATR Diamond/ZnSe ATR (single-reflection), was used to analyze the chemical functionalities of the specimens in the wavenumber range of 4000–650 cm^–1^ with four scans at a resolution of 4 cm^–1^. Three replicates of each specimen surface were collected by a microtome steel blade (Leica DB80 LX, Nussloch, Germany). X-ray photoelectron spectroscopy (XPS) analysis was carried out using a Kratos Axis Ultra DLD electron spectrometer equipped with a monochromated Al Kα source, operated at 150 W, with an analyzer pass energy of 160 eV for acquiring survey spectra and a pass energy of 20 eV for individual photoelectron lines being used. The surface potential was stabilized by the spectrometer charge neutralization system. The binding energy (BE) scale was referenced to the C 1s line of aliphatic carbon, set at 285.0 eV. Processing of the spectra was accomplished with the Kratos software. To avoid possible X-ray degradation effects, C 1s and O 1s spectra were acquired first within 10 min before the survey spectrum and spectra of minor elements. Three locations were analyzed on specimens of thickness 100 μm. The specimens were prepared by a Leica RM2255 rotary microtome equipped with a steel blade Leica DB80 LX (Nussloch, Germany). Solid-state ^13^C cross-polarization (CP) magic-angle-spinning (MAS), ^13^C CP-MAS, and the direct excitation ^31^P MAS NMR spectra, both with a high-power ^1^H-decoupling (spinal64, 90 kHz nutation frequency of protons), were obtained using “Varia” 4 and 5 mm MAS probes on a Bruker “Avance-III 400” NMR spectrometer with a superconducting “zero-helium boil-off” Ascend Aeon magnet (*B*_0_ = 9.4 T). The ^13^C/^31^P operating frequency was 100.64/162.01 MHz. Wood samples were cut into small pieces of ca. 1 × 1 × 1 mm in size and packed in zirconium dioxide standard double-bearing 4 or 5 mm Varian rotors (ca. 26/29 mg of the FR-modified wood before/after EN84, respectively, in a 4 mm rotor and ca. 73 mg of the unmodified wood in a 5 mm rotor). The proton π/2 pulse duration in the ^13^C CP-MAS NMR experiment was 3.3 μs, the CP mixing time was 2.0 ms and 25888 (FR-modified wood) or 4913 (unmodified wood) signal transients, spaced by the relaxation delay of 2.0 s, were accumulated. The ^31^P π/2 pulse duration in the direct excitation ^31^P MAS NMR experiments, all performed using the 4 mm MAS probe, was 4.1 μs, and (1198/1018) signal transients (for FR-modified wood before/after EN84, respectively), spaced by the relaxation delay of 10.0 s, were accumulated. The spinning frequency was 8000 ± 2 Hz in both ^13^C CP-MAS and ^31^P MAS NMR experiments. The isotropic ^13^C chemical shift (in the deshielding, δ-scale) was externally referenced to the least shielded resonance line of solid adamantane (at 38.48 ppm relative to tetramethylsilane).^103^^31^P chemical shift was externally referenced to 85% H_3_PO_4_ (0 ppm) filled in a glass capillary and placed in a ZrO_2_ 4 mm empty rotor to avoid large differences in the magnetic susceptibility between wood samples packed in ZrO_2_ rotors and the external liquid reference sample. All measurements were performed at ambient temperature (ca 295 K). Scanning electron microscopy (SEM), with a Jeol JSM-IT300LV (Tokyo, Japan) equipped with an energy-dispersive X-ray spectrometer in LUMIA (Luleå Material Imaging and Analysis), was utilized to investigate the morphology and the elemental composition of the specimens under a low-vacuum mode at 100 Pa through secondary electrons with the electron beam acceleration voltage set up at 10 and 15 kV, respectively. The working distance was set at approximately 10 mm for both SEM and EDX analyses. The spectrometer was eventually controlled by ZAtec V3.1 software (Oxford Instruments, Buckinghamshire, UK). The 100 μm thickness of the specimens was examined without any coating. Scanning times of about 300 s on three locations were applied for the EDX elemental mapping. pH value of the solutions was measured by a Metrohm pH meter 744 (Metrohm, Herisau, Switzerland). TGA was performed using a PerkinElmer TGA 4000. Specifically, 4 ± 1 mg of each specimen prepared by a microtome steel blade was loaded in an alumina crucible and heated from 30 to 800 °C with a rate of 10 °C min^–1^ under a N_2_ flow rate of 20 mL min^–1^. In total, three replicates were measured. The first derivative of the TGA curves was smoothed by a 20-point smoothing algorithm through the STARe Evaluation V16.20 software (Mettler Toledo, Columbus, Ohio, USA). The limiting oxygen index (LOI, Fire Testing Technology Ltd., East Grinstead, UK) test was conducted in accordance with ISO 4589-2:2017 using five replicates having a dimension of 10 × 10 × 150 mm (*T* × *R* × *L*).^104^ A cone calorimetry test was performed by the TCC 918 equipment (Netzsch, Selb, Germany) according to ISO 5660-1:2015 under a heat flux of 50 kW/m^2^ and using at least three replicates from the specimens measuring 100 × 10 × 100 mm (*T* × *R* × *L*).^105^ The heat release rate (HRR), the first peak in the HRR curve (pHRR_1_), the second peak in the HRR curve (pHRR_2_), the total heat release (THR), the smoke production rate (SPR), the total smoke production (TSP), the mass loss rate (MLR), and the time to ignition (TTI) were all recorded automatically by the cone calorimeter. The fire growth rate (FIGRA) was then calculated by dividing the value of pHRR_2_ by the time to reach pHRR_2_.^106^ The MARHE value was assessed by maximum value selection of the averaged HRR divided by its time interval.^107^ The prediction of the European fire classification of the specimens was based on a model of multivariate statistical analysis existing for a series of wood-based products.^92^ A criterion model 43 universal testing machine (MTS Systems Corporation, Créteil, France) was utilized for the four-point static bending test using 10 replicates. The modulus of elasticity (MOE) and the modulus of rupture (MOR) were measured in specimens having dimensions of 10 × 10 × 150 mm (*T* × *R* × *L*) in accordance with the EN 408:2012 standard.^108^ The calculation of MOE and MOR was made using eqs 6 and 7, respectively.
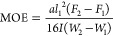
6
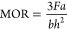
7where *a* is the distance between the loading position and the nearest support, *b* is the width of the specimen, *h* is the thickness of the specimen, *l*_1_ is the gauge length of the span, *I* is the second moment of area, *F* is the load, *F*_2_^1^*F*_1_ is the increment of the load between 40 and 10% of *F*, and *W*_2_–*W*_1_ is the increment of deformation corresponding to *F*_2_^1^*F*_1_, respectively.

## Supporting Information Available

Photographs of the unmodified wood and the FR-modified wood and color measurements; changes in the pH of the leached water during EN 84 testing; the SEM–EDX elemental mapping images of the FR modified wood for carbon, oxygen, nitrogen, and phosphorus; SEM–EDX elemental mapping images of the FR modified wood after water-leaching EN84 for carbon, oxygen, nitrogen, and phosphorus (PDF)

## Abbreviations

ADP: ammonium dihydrogen phosphate
BC: bulking coefficient
CP-MAS NMR: cross-polarization magic-angle-spinning nuclear magnetic resonance
DMDHEU: dimethylol dihydroxyethylene urea
DSC: differential scanning calorimetry
DI: deionized
DTG: derivative thermal gravimetry
EDX: energy dispersive X-ray spectrometry
EMC: equilibrium moisture content
FIGAR: fire growth rate
FTIR: Fourier transform infrared
FR: fire-retardant
GUP: guanyl-urea phosphate
HMF: 5-hydroxymethylfurfural
HRR: heat release rate
*L*: longitudinal
LOI: limiting oxygen index
MARHE: maximum average rate of heat emission
MC: moisture content
MF: melamine-formaldehyde
MOE: modulus of elasticity
MOR: modulus of rupture
PF: phenol-formaldehyde
*R*: radial
RH: relative humidity
SEM: scanning electron microscopy
SPR: smoke production rate
*T*: tangential
TGA: thermal gravimetric analysis
THR: total heat release
TSP: total smoke production
TTI: time to ignition
WPG: weight percentage gain
XPS: X-ray photoelectron spectroscopy
