# Supplementary material for: High Leach-Resistant Fire-Retardant Modified Pine Wood (Pinus sylvestris L.) by In Situ Phosphorylation and Carbamylation
Source: ACS Omega. 2023 Mar 14;8(12):11381–96. doi: 10.1021/acsomega.3c00146 (PMC10061617; doi:10.1021/acsomega.3c00146)
Supplement: Supplementary file 1 — ao3c00146_si_001.pdf [file ao3c00146_si_001.pdf]

High Leach-Resistant Fire-Retardant Modified Pine Wood (*Pinus sylvestris* L.) by *in-situ* Phosphorylation and Carbamylation

Chia-feng Lin[*a], Olov Karlsson[a], Oisik Das[b], Rhoda Afriyie Mensah[b], George I. Mantanis[c], Dennis Jones[a, d], Oleg N. Antzutkin[e], Michael Försth[b] and Dick Sandberg[a, d]*

[a]Wood Science and Engineering, Department of Engineering Sciences and Mathematics, Luleå University of Technology, Forskargatan 1, SE-931 77 Skellefteå, Sweden

[a]Structural and Fire Engineering, Department of Civil, Environmental and Natural Resources Engineering, Luleå University of Technology, SE-971 87 Luleå, Sweden

[c]Laboratory of Wood Science and Technology, Department of Forestry, Wood Sciences and Design, University of Thessaly, GR-431 00 Karditsa, Greece

[d]Department of Wood Processing and Biomaterials, Faculty of Forestry and Wood Sciences, Czech University of Life Sciences Prague, Praha 6-Suchdol, CZ-16521 Prague, Czech Republic

[e]Chemistry of Interfaces, Department of Civil, Environmental and Natural Resources

Engineering, Luleå University of Technology, SE-971 87 Luleå, Sweden

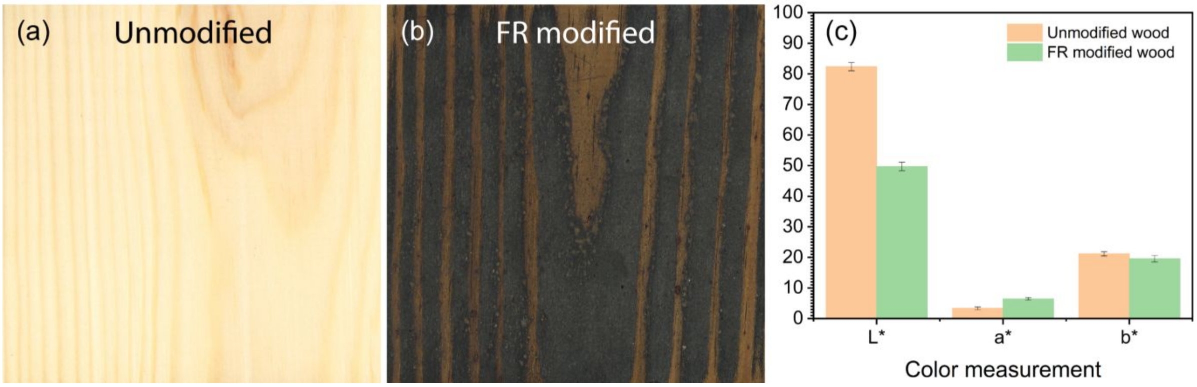

Figure S1. Photographs of (**a**) the unmodified wood and (**b**) the FR-modified wood and (**c**) color measurements.

Table S1. Changes in pH of the leached water during EN 84 testing.

Day	0	1	2	4	6	7	9	11	13	14
pH	3.33	3.71	3.92	3.94	4.10	4.43	4.46	4.45	4.66	4.94

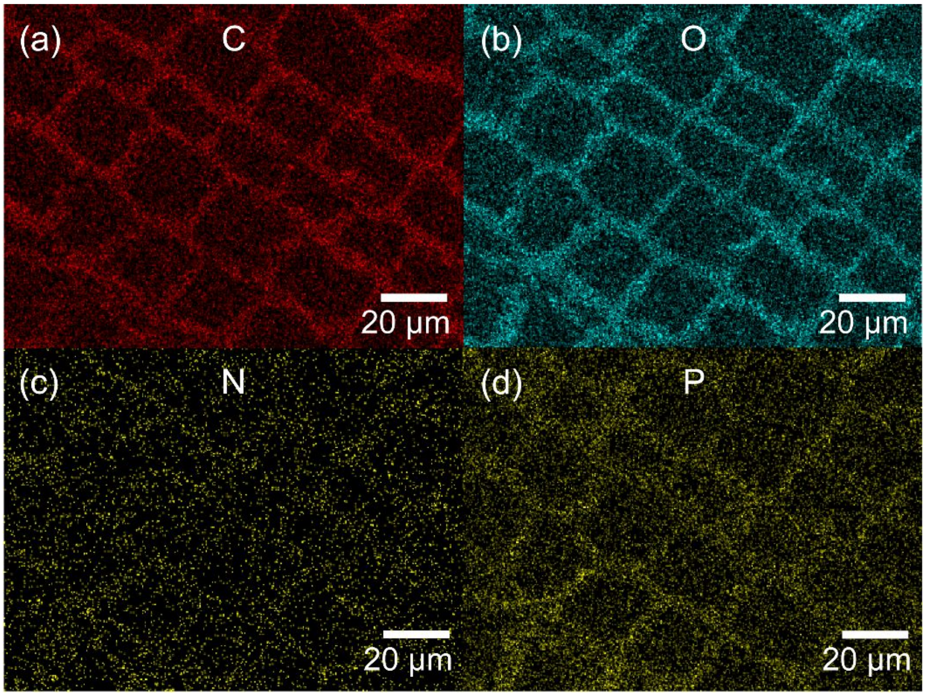

Figure S2. The SEM-EDX elemental mapping images of the FR modified wood for: (**a**) carbon, (**b**) oxygen, (**c**) nitrogen and (**d**) phosphorus, respectively.

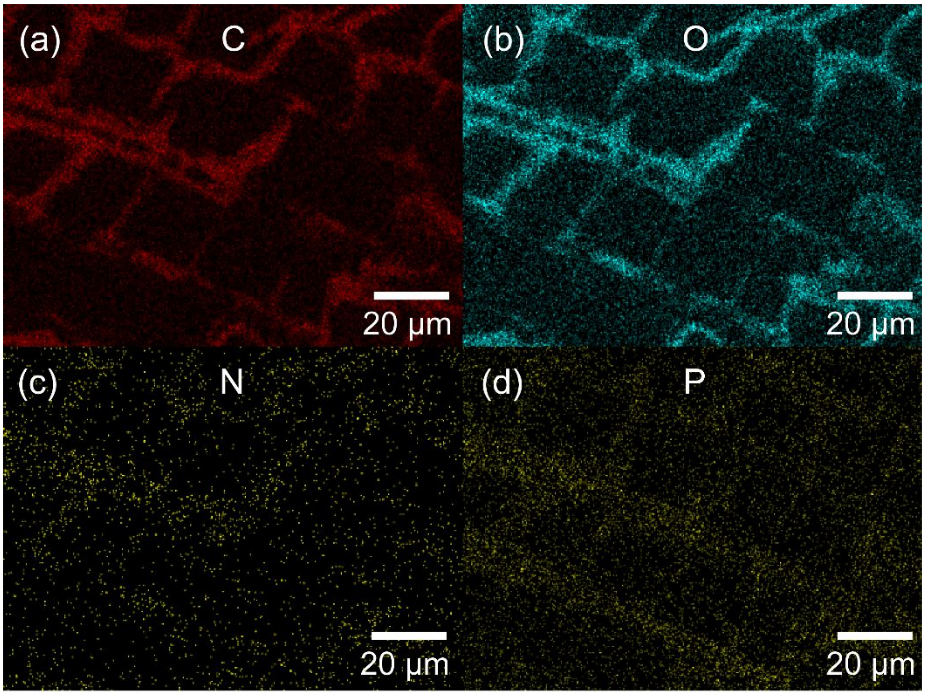

Figure S3. The SEM-EDX elemental mapping images of the FR modified wood after water-leaching EN84 for: (**a**) carbon, (**b**) oxygen, (**c**) nitrogen and (**d**) phosphorus, respectively.
